# Editorial: Comparative Endocrine Stress Responses in Vertebrates

**DOI:** 10.3389/fendo.2019.00652

**Published:** 2019-09-24

**Authors:** Lluis Tort, John F. Cockrem, Edward J. Narayan

**Affiliations:** ^1^Department Cell Biology, Physiology and Immunology, Universitat Autonoma de Barcelona, Barcelona, Spain; ^2^School of Veterinary Science, Massey University, Wellington, New Zealand; ^3^School of Science and Health, Western Sydney University, Penrith, NSW, Australia; ^4^School of Agriculture and Food Sciences, The University of Queensland, Gatton, QLD, Australia

**Keywords:** editorial, stress, responses, endocrine, vertebrates

The stress response in vertebrates is characterized by involving physiological regulatory systems and a number of organs, tissues, and effector pathways in order to both respond to the stressor effects and overcome the situation and recover homeostasis. Although differences in specific mechanisms are encountered in different animal groups and even between interspecies and intraspecies, the stress response involves an endocrine activation in all groups of vertebrates. Since an increasing number of scientific works are currently published in this field, the idea of collecting and reviewing such advances originated the initiative of a Topic Collection on the Comparative Stress responses in Vertebrates. In the following set of papers, the readers will find out an interesting update of the latest work in the field of the endocrine responses to stress in vertebrates, from general approaches to specific contributions and methodological updates.Of course, it is not intended that this *Topic* collection be exhaustive or complete, as many untreated aspects could be added, and some vertebrate groups are not well-represented into the collection, but this overview is anyway interesting to show what are some of the current working areas in this field.

Among the general approaches presented in this Topic collection, a novel contribution is the concept of *stressotope* by Balasch and Tort, linking the adaptive set of responses of the animal to particular biotopes associated with specific conditioning factors involving the maximum overall stress responses across immune-neuroendocrine relevant physiological levels and scenarios, including the characterization of behavioral response.

In relation with this stressotope concept, the work by Sánchez-Vázquez et al., deep into the relationship among factors regulating the circadian rhythms in animals particularly under stress situations, showing that not only a number of specific environmental factors are connected to circadian rhythms, but also that the proper oscillations of the environmental factors are significantly involved.

In another of the works in this *Topic*, Gómez-Boronat et al., investigating in daily cycles, demonstrate that the misalignment of external cues such as day-night photocycles and feeding time may temporarily alter fish homeostasis, thus involving a stress situation for the animals.

Other contributions make relevant insights in the comparative approach of endocrine stress regulation in vertebrates. In the first one, Narayan and Vanderneut provided invaluable insights into how wild koalas respond physiologically to environmental trauma and disease, a species not often represented in the scientific literature of comparative stress responses. In addition, this paper includes an applied aspect on how methods of care, husbandry, and treatment can be used to reduce the impacts of stressors with the ultimate aim of increasing the rehabilitation possibilities and future release of this species in the wild.

In another paper, Höglund et al. show the role of Tryptophan and the associated metabolic pathways in the regulation of serotonergic activity in the fish brain, a key mechanism intimately associated to stress and behavioral responses of animals. Numerous studies have shown that elevated dietary Trp has a suppressive effect on aggressive behavior and post-stress plasma cortisol concentrations in vertebrates. These effects are believed to be mediated by the brain serotonergic system, even though mechanisms involved are not well-understood.

Also regarding key components in the diet, the work by Herrera et al., have looked at the studies on stress attenuation in animals through diet or supplement components. Other than the development of new technologies to monitor and improve environmental conditions of farmed animals, particularly fish, beneficial additives in the daily meal have been included in order to mitigate the effects of husbandry stressors. Immunological, nutritional, and metabolic changes have been assessed in these trials, always associated to endocrine regulation. The biochemical and physiological functionality of those feed additives may strongly affect the stress response and, even, such additives may act as neurotransmitters, hormone precursors, energy substrates, or cofactors implying multi-systematic and multi-organic responses that modify the response to stress.

Suarez-Bregua et al. focused their approach in a less studied area in lower vertebrates as the endocrine relationship between glucocorticoid metabolism and the parathyroid hormone family peptides. The paper deeps into the response driven by these hormones and other key regulators of mineral homeostasis in connection with bone remodeling processes, which involves important consequences in terms of harmonic growth and skeletal deformities.

A more specific comparative work on the stress and endocrine responses is presented by a group of researchers from Greece, Norway, and The Netherlands. Thus, Samaras et al. focus on the differential responses between two close warm water marine aquacultured species, sea bass, and sea bream. In this paper, they show how significant can be the species-specific molecular and neuro-regional differences between two similar species sharing many environmental and geographical conditions. This points out how important can be the variability of specific mechanisms between species, even from close-related groups, though sharing the basic patterns of molecular and endocrine molecules and pathways.

Finally, regarding key methodological contributions, the paper by Aerts rises the importance of the methodological aspects associated to the molecules currently chosen to define a stressed status. He demonstrates that it is pivotal to know the involved regulatory molecules and to understand how these molecules are synthesized, regulated, and excreted, together with how these molecules grasp their actions on a plethora of biological processes in many organs and tissues.

Collectively, the *Topic* highlights current research areas and future directions in the dynamic field of vertebrate stress endocrinology. Beyond theoretical knowledge, the field of research provides powerful tools to enable researchers to make objective assessments of the physiological state of animals, to understand how animals respond to environmental change and human interventions.

## Author Contributions

All authors listed have made a substantial, direct and intellectual contribution to the work, and approved it for publication.

### Conflict of Interest

The authors declare that the research was conducted in the absence of any commercial or financial relationships that could be construed as a potential conflict of interest.

